# The Macromolecular Femtosecond Crystallography Instrument at the Linac Coherent Light Source^1^

**DOI:** 10.1107/S1600577519001577

**Published:** 2019-02-22

**Authors:** Raymond G. Sierra, Alexander Batyuk, Zhibin Sun, Andrew Aquila, Mark S. Hunter, Thomas J. Lane, Mengning Liang, Chun Hong Yoon, Roberto Alonso-Mori, Rebecca Armenta, Jean-Charles Castagna, Michael Hollenbeck, Ted O. Osier, Matt Hayes, Jeff Aldrich, Robin Curtis, Jason E. Koglin, Theodore Rendahl, Evan Rodriguez, Sergio Carbajo, Serge Guillet, Rob Paul, Philip Hart, Kazutaka Nakahara, Gabriella Carini, Hasan DeMirci, E. Han Dao, Brandon M. Hayes, Yashas P. Rao, Matthieu Chollet, Yiping Feng, Franklin D. Fuller, Christopher Kupitz, Takahiro Sato, Matthew H. Seaberg, Sanghoon Song, Tim B. van Driel, Hasan Yavas, Diling Zhu, Aina E. Cohen, Soichi Wakatsuki, Sébastien Boutet

**Affiliations:** a Linac Coherent Light Source, SLAC National Accelerator Laboratory, Menlo Park, CA 94025, USA; b School of Physical Science and Technology, ShanghaiTech University, Shanghai 201210, People’s Republic of China; c Stanford Synchrotron Radiation Lightsource, SLAC National Accelerator Laboratory, Menlo Park, CA 94025, USA; d BioSciences Division, SLAC National Accelerator Laboratory, Menlo Park, CA 94025, USA; e PULSE Institute, SLAC National Accelerator Laboratory, Stanford University, Menlo Park, CA 94025, USA; f Brookhaven National Laboratory, Upton, NY 11973, USA; g Department of Structural Biology, School of Medicine, Stanford University, Stanford, Menlo Park, CA 94305, USA

**Keywords:** X-ray free-electron laser, serial femtosecond crystallography, protein crystallography, time-resolved crystallography, room-temperature crystallography, X-ray FEL

## Abstract

The Macromolecular Femtosecond Crystallography (MFX) instrument at the Linac Coherent Light Source (LCLS) is the seventh and newest instrument at the world’s first hard X-ray free-electron laser. It was designed with a primary focus on structural biology, employing the ultrafast pulses of X-rays from LCLS at atmospheric conditions to overcome radiation damage limitations in biological measurements. It is also capable of performing various time-resolved measurements. The MFX design consists of a versatile base system capable of supporting multiple methods, techniques and experimental endstations. The primary techniques supported are forward scattering and crystallography, with capabilities for various spectroscopic methods and time-resolved measurements. The location of the MFX instrument allows for utilization of multiplexing methods, increasing user access to LCLS by running multiple experiments simultaneously.

## Abstract 2

A description of the Macromolecular Femtosecond Crystallography instrument at the Linac Coherent Light Source is given. Scientific capabilities are highlighted, performance parameters are presented along with some commissioning results.

## 1. Introduction   

The Macromolecular Femtosecond Crystallography (MFX) instrument is located in the newest experimental hutch at the Linac Coherent Light Source (LCLS) (White *et al.*, 2015 ▸), where a space between the fourth and fifth instruments was re-purposed into what is now known as Hutch 4.5. The MFX instrument was built primarily to exploit the ultrashort pulses from the LCLS X-ray free-electron laser (FEL) to minimize or overcome the adverse effects of radiation damage in structural biology measurements using X-rays (Neutze *et al.*, 2000 ▸). Specifically, the MFX instrument aims to provide a versatile atmospheric-pressure system for both static and time-resolved macromolecular crystallography. Time-resolved measurements are possible using excitation mechanisms such as optical lasers or chemical mixing, methods that have been developed over the first decade of X-ray FEL operations, as described in a recent book (Boutet *et al.*, 2018 ▸).

The MFX instrument complements the capabilities of the Coherent X-ray Imaging (CXI) instrument (Liang *et al.*, 2015 ▸) which is also commonly used for structural biology. MFX provides an atmospheric-pressure sample environment instead of the vacuum environment of the CXI instrument. The atmospheric-pressure environment provides increased flexibility and ease of operation compared with CXI and duplicates the previously existing capability of the X-ray Pump–Probe (XPP) instrument (Chollet *et al.*, 2015 ▸) to deploy multiple endstations at atmospheric pressure. MFX provides the advantage of a more dedicated and accessible system for easier deployment (Boutet *et al.*, 2016 ▸). The MFX instrument provides flexible X-ray focusing capabilities to tailor the X-ray spot size to the sample. The instrument consists of a versatile base system with a large breadboard and two detector mounting mechanisms that can support multiple existing experimental geometries and endstations, while allowing for future technological developments. The MFX instrument can support other experimental techniques beyond macromolecular crystallography applications; such as solution scattering or emission spectroscopy, for example.

MFX experiments can take advantage of multiplexing techniques, increasing the overall number of biological experiments performed at LCLS, by using the same X-ray beam at multiple instruments at the same time. The location of the instrument in the Far Experimental Hall (FEH) of LCLS allows MFX to use the transmitted beam from the XPP instrument located upstream, increasing access to beam time (Feng *et al.*, 2015 ▸) by increasing the amount of simultaneous experiments. Currently, roughly 25% of LCLS experiments are multiplexed and this fraction is expected to grow. Historically, 33% of all LCLS experiments have been in the life science field and making these experiments compatible with multiplexing provides a path to grow access.

The motivation behind the addition of the MFX instrument to the LCLS complex was previously presented (Boutet *et al.*, 2016 ▸). Here, the focus is on the technical components and capabilities of the instrument, along with commissioning results and scientific use of the instrument, as well as recent results from the user community.

## 2. Instrument overview   

The MFX instrument operates in the hard X-ray regime (*i.e.* above 5 keV or ≲2.5 Å). A summary of the instrument parameters is available in Table 1 ▸. MFX is installed on the hard X-ray branch of LCLS which includes a pair of mirrors in the LCLS Front-End Enclosure (FEE). These mirrors were recently upgraded in 2017. They are 1 m long — much longer than the previous mirrors — with two coatings to allow for harmonic rejection over the range of usable photon energies produced by LCLS. The two coatings are B_4_C and Ni and operate at incidence angles of 2.1 mrad, leading to high-energy cutoffs of ∼13 keV and ∼26 keV, respectively. The extra length provides a clear aperture sufficiently large to accept the whole LCLS beam with minimal wavefront distortions to produce a mostly round beam for the experiments, as recently characterized (Liu *et al.*, 2018 ▸). These mirrors were designed with the upcoming upgrade to LCLS-II in mind, which will deliver up to 25 keV at the fundamental energy using the existing LCLS copper accelerator with a new variable-gap undulator. Therefore, when LCLS-II turns on in 2020, the fundamental energy range usable at MFX will extend up to 25 keV (Galayda, 2014 ▸).

**Figure 1:**
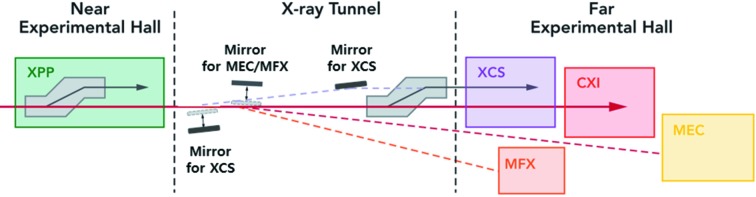
A schematic of the LCLS hard X-ray experimental hutches and their beam distribution system. Not shown are two mirrors upstream (left) of the XPP hutch which form a periscope to deliver the beam to XPP and beyond. Two large-offset double-crystal monochromators (LODCMs) can deliver monochromatic beam to the X-ray Pump–Probe (XPP) and X-ray Correlation Spectroscopy (XCS) instruments while transmitting most of the pink beam through the first crystal to another downstream instrument. One LODCM is located at XPP and the second one is near the end of the X-Ray Tunnel. Three mirrors in the X-Ray Tunnel can deliver the pink beam to all hutches of the Far Experimental Hall on their own beamline. Two mirrors form a periscope to XCS. The mirror for MEC/MFX can be used at two different angles to deliver the beam to the desired hutch. All mirrors are removed from the beam path to allow the beam to reach CXI. The MFX instrument can perform pink-beam experiments when multiplexed with the XPP instrument using the first LODCM in the Near Experimental Hall. The diagram illustrates that, although CXI and MEC are downstream of MFX, they are on differing paths and cannot be multiplexed with MFX in the typical way of using a thin crystal.

A similar mirror is located downstream of the LCLS Near Experimental Hall (NEH) in the X-Ray Tunnel (XRT) (White *et al.*, 2015 ▸). This mirror can send the beam to either the Matter in Extreme Conditions (MEC) instrument (Nagler *et al.*, 2015 ▸) or the MFX instrument at twice the deflection angle compared with MEC (Boutet *et al.*, 2016 ▸), see Fig. 1 ▸. This mirror was also upgraded in 2017 to have a larger acceptance and surface quality. All upgraded hard X-ray mirrors can accept the larger beam at photon energies down to ∼3 keV. The low-energy cutoff of the MFX instrument is not driven by the beam distribution mirrors but rather by the operation in air and the efficiency of the beryllium compound refractive lenses used for focusing, setting ∼5 keV as the practical lower limit. The XRT mirror has two coating strips of B_4_C and Rh operating at 2.75 mrad which leads to high-energy cutoffs of ∼11 keV and ∼22 keV, respectively. Although the MFX instrument is located upstream of CXI and MEC, the beam cannot be multiplexed past MFX, due to the mirror geometries seen in Fig. 1 ▸. MFX can only currently multiplex the beam with XPP.

**Figure 2:**
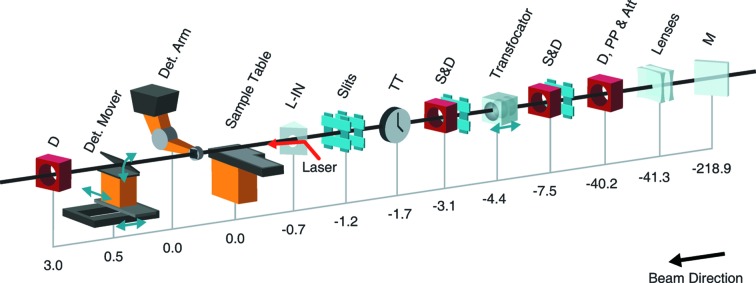
Overview of the MFX instrument layout. Distances are indicated in meters from the nominal interaction region on the sample table (with the detector arm also sitting at zero, directly above the sample table); positive values indicate the direction of beam propagation. M is a mirror located in the LCLS X-ray Tunnel (XRT) with two coating stripes to provide tunable harmonic rejection and to deflect the beam to MFX. Lenses at −41.3 are pre-focusing compound refractive lenses. D, PP & Att at −40.2 is a diagnostic section which includes a Ce:YAG screen for beam viewing, a single-pulse picker and a set of ten independent silicon attenuators of various thicknesses. S&D are slits, a beam-viewing YAG screen diagnostic and a non-destructive intensity measurement diagnostic. A transfocator system is used to mount ten independent stacks of compound refractive lenses to produce a controllable spot size at the sample. TT is a timetool measuring the arrival time of the optical laser in reference to the X-rays (not yet available at MFX but expected in the future). The slits at location −1.2 are double slits to allow for blocking the harmonics of the beam. L-IN is the laser in-coupling for the optical laser. D at 3.0 is a beam-viewing Ce:YAG screen diagnostic to view the beam after it passes through the hole in the detector. The sample at the MFX instrument (0.0 on this figure) is located approximately 420 m downstream of the X-ray source which is within the undulator.

The MFX instrument utilizes the full bandwidth of the beam generated by the LCLS lasing process (commonly known as pink beam). This not only allows the use of the full spectrum of the self-amplified spontaneous emission (SASE) beam, but also various two-color (Marinelli *et al.*, 2015 ▸) and seeding methods (Amann *et al.*, 2012 ▸). There are no monochromators on the MFX beamline. After deflecting off the two mirrors in the FEE and the third mirror in the XRT, the beam first encounters devices on the MFX beamline ∼370 m downstream of the source. Fig. 2 ▸ shows the schematic layout of the MFX components.

### 2.1. Slits and diagnostics   

MFX utilizes four sets of slits to reduce errant background signals from upstream scatter. The first set — located 3.1 m upstream of the transfocator — removes the portion of the X-ray beam that is larger than the clear aperture of the focusing lenses. The next three sets of slits clean the beam after the focusing optics, reducing errant background scatter even further. The first three slits are made of Si_3_N_4_ which can withstand the full LCLS beam, at 120 Hz, without damage in the 5–25 keV range of capable operation at MFX (at the time of publication LCLS can produce up to 13 keV at the fundamental energy; however, LCLS-II upgrades will allow up to 25 keV). The fourth slits (part of a dual slit at location −1.2 in Fig. 2 ▸) use a Ta/W alloy capable of stopping the harmonics of the beam and up to 25 keV efficiently. Multiple diagnostics are available along the instrument, including beam profile monitors and intensity–position monitors (Feng *et al.*, 2011 ▸). Ten independently movable silicon attenuators, each on their own actuator, ranging in thickness from 20 µm to 10.24 mm are available in the XRT to control beam intensity. The finite number of solid attenuators can be combined to create discrete values of attenuation, depending on the incident X-ray wavelength.

### 2.2. Pulse picker   

A fast shutter can be used to select single X-ray pulses on demand or for generating a regular repetition rate slower than the source’s typical rate of 120 Hz. A rotating channel allows for an opening to let the beam pass for a duration shorter than the spacing between pulses. The device, known as a pulse picker, can provide an arbitrary pulse pattern up to a maximum of 30 Hz peak pulse rate, limited only by the oscillating speed of the pulse picker’s drive motor.

### 2.3. Transfocator and pre-focusing   

The MFX instrument uses compound refractive lenses (CRLs) made of beryllium to focus the beam at the sample or control the spot size at the sample. At MFX, the nominal distance from the focusing lenses to the sample is 4.4 m as shown in Fig. 2 ▸. The unfocused beam at this location is on the order of 1 mm × 1 mm, while the clear aperture of CRLs with a ∼4 m focal length at hard X-ray energies is typically on the order of 400 µm. This mismatch inefficiently transmits the beam to the sample; this can be mitigated via pre-focusing the beam. MFX therefore uses a set of pre-focusing lenses located 36.9 m upstream of the transfocator in the XRT which allows the beam to better match the main focusing lens aperture. The MFX pre-focusing lenses have radii of 750 µm (2 × 1500 µm, bottom stack), 429 µm (2 × 1500 µm + 1 × 1000 µm, middle stack) and 333 µm (3 × 1000 µm, top stack). These pre-focusing lenses have varying apertures depending on their radius of curvature but typically have >1.5 mm clear apertures and transmissions above 80% over the usable photon energy range for MFX.

The MFX main focusing lens system consists of ten independent actuators each containing a different number of lenses representing different effective radii of curvature. This system is known as a transfocator (Vaughan *et al.*, 2011 ▸) and overcomes the limitations of chromatic optics such as CRLs and allows for all current and future LCLS and LCLS-II hard X-ray energies (5 to 25 keV with no energy gaps in coverage) to be focused at the sample location with the use of a ±150 mm stage to translate the lenses along the beam axis (*Z* direction). The lens stacks in the MFX transfocator have radii of 500 (1 × 500), 300 (1 × 300), 250 (2 × 500), 200 (1 × 200), 125 (4 × 500), 62.5 (8 × 500), 50 (10 × 500), 50 (6 × 300) and 50 (4 × 200) µm for stacks number 2 to 10, respectively, with the first actuator left empty for a potential pinhole. The lenses are arranged from larger radius upstream (weaker lens) to smaller radius downstream (stronger lens) in order to minimize the losses from the lens apertures. The lenses are mounted in the center of each holder with spacers to fill the holder. For the no-pre-focus situation, transmission at 6–9.5 keV can be estimated at 25–35% for the installed lenses. Combined with the pre-focusing lenses, the MFX optical system provides both flexibility and efficiency. Commissioning results are presented in Section 5.

### 2.4. Sample table   

**Figure 3:**
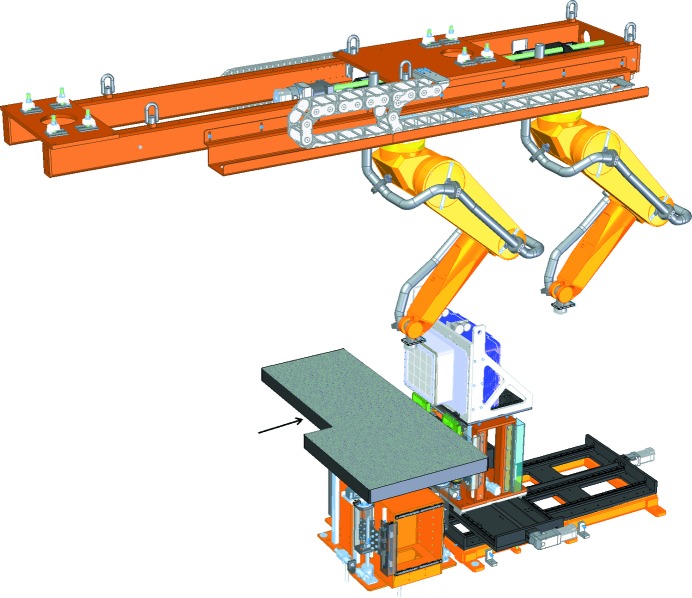
Design of the MFX endstation base system. It includes a ceiling-mounted detector robot arm which provides the degrees of freedom necessary to position the detector within a ∼2 m-diameter sphere. The robot arm is installed on a large translation stage that can move along the beam axis. The single robot is shown here in its two extreme locations. The MFX endstation base system also includes a large breadboard sample table with six degrees of motion and a detector mover for large area detectors in forward-scattering geometry. The detector mover is capable of tilting the detector up to 30° from horizontal. It is shown here with the Rayonix MX340-XFEL mounted on it. The arrow indicates the beam direction.

The MFX instrument provides a flexible experimental setup, enabling a multitude of experimental geometries, focused primarily on macromolecular X-ray crystallographic studies. The sample environment sits atop a large breadboard with six degrees of motion, which provides a large open geometry to support a variety of purpose-specific endstations easily mounted on an optical breadboard as shown in Figs. 3 ▸ and 4 ▸. The sample table is capable of moving 508 mm horizontally transverse to the beam (*X*), 216 mm vertically (*Y*) and 172 mm horizontally along the beam (*Z*). Pitch, yaw and roll can be adjusted to level and align equipment mounted to the table.

**Figure 4:**
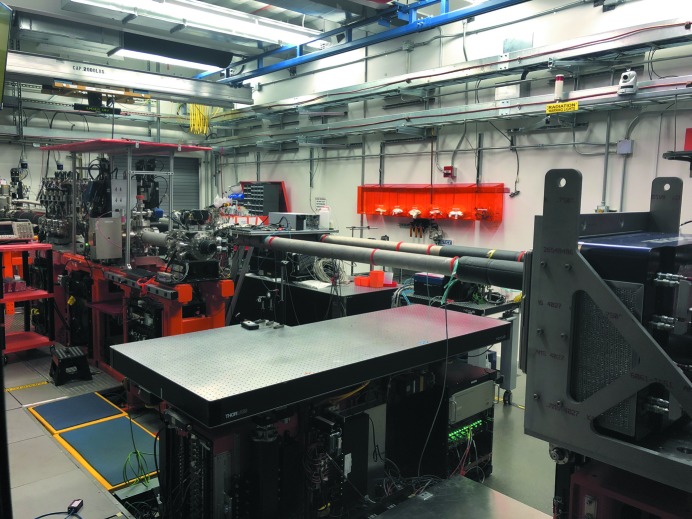
The MFX hutch as seen from the southeast corner. The unfocused beam enters from the left of the image, and propagates through diagnostics and slits, and continues to the transfocator and further on through more diagnostics and slits. The focused beam exits a diamond window to atmosphere towards the large breadboard of the sample table, seen here empty. To the right is the Rayonix MX340-XFEL detector on the detector mover. Above the table (not seen) is the robot arm, which can also be equipped with another smaller detector. The beam transport pipes for the MEC and CXI instruments can be seen adjacent to the MFX table.

Typical endstations used at MFX are: a goniometer-based system with an automated sample exchange robot developed at the Stanford Synchrotron Radiation Lightsource (SSRL) (Cohen *et al.*, 2014 ▸), a droplet-on-tape system developed by a team from Lawrence Berkeley National Laboratory (Fuller *et al.*, 2017 ▸), a 120 Hz chip-scanning system from the Center for Free-Electron Laser Science (CFEL) (Roedig *et al.*, 2017 ▸) and a helium-filled enclosure for liquid jets developed by the Max Planck Institute for Medical Research in Heidelberg, similar to the Diverse Application Platform for Hard X-ray diffractioN In SACLA (DAPHNIS) system at the SPring-8 Angstrom Compact Free-Electron Laser (SACLA) (Tono *et al.*, 2015 ▸). These all interface easily to the MFX sample table and will be described in more detail in Section 3. A helium-filled flight tube is used to deliver the final, focused beam to the experiment with minimal stray scattering and connects the beamline, from a diamond window letting the X-ray beam out of vacuum, to the endstation of choice on the sample table. The optical breadboard interface of the sample table also makes it capable of laser transport and in-coupling for time-resolved optical pump–probe experiments. The table was designed to interface with the SSRL goniometer and sample robot system, to which it owes its non-rectangular shape. Other systems were made to adapt to the sample table or fortuitously fit without modifications. The beam transport pipes delivering the beam to the CXI and MEC instruments (visible in part on Fig. 4 ▸) must pass through the MFX hutch and represent a limitation to the size in the transverse horizontal direction for systems to be installed on the MFX table.

### 2.5. Laser systems   

Currently, MFX can utilize only nanosecond pulse duration lasers, including Nd:YLF nanosecond laser systems (Young *et al.*, 2016 ▸; Kupitz *et al.*, 2014 ▸) and a tunable Optical Parametric Oscillator (OPO) system capable of 200–2200 nm wavelength generation, with 8 ns pulse duration. The MFX instrument currently does not have a dedicated ultrafast femtosecond laser system but plans exist to add this capability by early 2020. This femtosecond laser system will consist of an ultrashort-pulse Ti:sapphire oscillator synchronized to the FEL seeding a commercially available chirped pulse amplifier producing 4 mJ at 40 fs. A more in-depth description of the optical laser capabilities at LCLS has been given by Minitti *et al.* (2015 ▸). The arrival time of the femtosecond laser will then be measured using a standard LCLS timetool (Harmand *et al.*, 2013 ▸) to be made available at the same time as the femto­second laser system.

### 2.6. Detectors   

#### 2.6.1. Detector mover   

Large area detectors in the forward-scattering geometry are a critical part of any macromolecular crystallography experiment. To position such a detector, MFX is equipped with a detector mover with micrometer positioning accuracy to center the detector on the incident X-ray beam. The detector mover has over 1 m of travel along the beam axis (*Z*) and 350 mm horizontally transverse to the beam (*X*). It is also equipped with two vertical stages that allow the detector and its potential central hole to be aligned to the beam and also tilted up to 30° around a pivot axis to allow for higher-resolution measurements. The detector mover is built to mount large heavy detectors such as the Rayonix MX170-XFEL and a recently deployed MX340-XFEL (Rayonix, LLC; Evanston, IL, USA), which are both equipped with central holes to let the direct beam pass through safely. The presence of a central hole in the detector removes the necessity for a beamstop that is typically used to minimize the path that X-rays travel through air that has a line of sight to the detector. Such an air path creates background scattering on the detector. The beamstop can be replaced by a hollow metal tube inserted in the detector hole and can be brought close to the interaction region. All air scatter from within this tube is absorbed by the tube material which reduces the background on the detector, while still allowing the main X-rays to safely propagate through.

#### 2.6.2. Detector robot   

A ceiling-mounted robot arm identical to the system in use at the XPP instrument is available for other detector positioning possibilities, such as to measure X-ray emission signals or to place a detector at a high scattering angle but far from the sample to perform Bragg coherent diffractive imaging measurements, for example (Clark *et al.*, 2013 ▸). The robot has six degrees of freedom allowing for the detector to be positioned within a ∼2 m-diameter sphere while facing the interaction region. The detector can also face any direction for special needs. The detector robot is capable of carrying relatively light detector systems (<25 kg) such as the Cornell–SLAC Pixel Array Detector CSPAD 2.3M (2.3 megapixels) and its smaller counterpart the CSPAD 140K (140000 pixels). It is also used for mounting ePix100 detectors (Carini *et al.*, 2016 ▸) and future members of the ePix detector family being developed at LCLS and SLAC such as the ePix10K (Blaj *et al.*, 2019 ▸), which will provide up to 10000 X-ray photons per pixel readout with single-photon sensitivity. It can also be used to support Jungfrau 0.5M and 1M detectors, which are now available at LCLS.

#### 2.6.3. Detector selection   

The optimal repetition rate for data collection at MFX is governed by the detector chosen and the pulse repetition rate. The current maximum data rate is achieved using the atmospheric Cornell-SLAC Pixel Array Detector (CSPAD) 2.3M (Hart *et al.*, 2012 ▸) with no pulse picker, *i.e.* operating at the source’s maximum rate of 120 Hz. This can be increased in the future with detector and source upgrades. The Rayonix detectors work at slower repetition rates but offer higher dynamic range than the high-sensitivity, single-photon-sensitive CSPAD. The Rayonix detectors can be binned in order to achieve faster data readouts; however, binning too many pixels together makes accurate measurement of the intensity and position of the Bragg reflections difficult, as they will be averaged with adjacent noise. The Rayonix MX340-XFEL is typically operated in 4 × 4 binning mode, at a maximum rate of 30 Hz (1920 × 1920 pixels, 177 µm pixel size) or in 3 × 3 binning mode, at a maximum rate of 20 Hz (2560 × 2560 pixels, 133 µm pixel size). Note that it is possible to bin the pixels further, creating larger effective pixels, to achieve higher detector acquisition rates than 30 Hz. Beyond the natural operation at a full 120 Hz rate, 60 Hz is also possible with both the CSPAD and appropriate binning of the Rayonix detectors, but the 60 Hz rate can only be achieved by reducing the repetition rate at the source due to the MFX pulse picker being physically limited to a maximum of 30 Hz. Operating MFX between 30 and 120 Hz is not typically possible since MFX is multiplexed most of the time with an XPP experiment using the full 120 Hz repetition rate of LCLS. The MFX pulse picker is the only way to reduce the rate of pulses from the source rate upstream.

All available detectors have tradeoffs: some have a very high dynamic range but lack single-photon sensitivity while others operate at the full current rate of LCLS of 120 Hz but have less than ideal noise features, complex geometries, too few pixels or lack a central hole. Selecting the correct detector for the experiment is one of the critical decisions that needs to be made with an instrument scientist. A data-hungry time-resolved experiment might benefit from the higher repetition rate of the CSPAD, but would then be more sensitive to background noise due to the smaller dynamic range, possibly requiring a helium environment to reduce air scatter. Conversely, an in-air or viscous media injection might require the dynamic range afforded by the Rayonix detectors, albeit at slower data rates. Overall, the combination of all detectors available at LCLS and the two detector positioning systems makes MFX ready to support a broad range of experimental techniques.

## 3. Spectrometers   

The spectrum of the incident FEL SASE pulse can be measured by a thin bent crystal transmissive spectrometer located in the FEE or downstream of the sample (Zhu *et al.*, 2012 ▸). A suite of X-ray emission spectrometers, namely multicrystal X-ray emission spectrometers based on the wavelength-dispersive von Hamos geometry (Alonso-Mori *et al.*, 2012 ▸) and on the point-to-point Rowland geometry, are available to be deployed at the MFX instrument. These spectrometers provide the capability to collect photon-in–photon-out spectra either independently or simultaneously to other forward-scattering techniques. While the simultaneous use of diffraction and spectrocopy is increasing, limited published work exists to date from MFX. Examples of such simultaneous measurements have been published from the CXI instrument (Kern *et al.*, 2013 ▸) and a similar geometry is now available at MFX (Fuller *et al.*, 2017 ▸; Kern *et al.*, 2018 ▸). Typically, the diffraction detector is positioned on the detector mover, the analyzer crystals of the spectrometer are mounted to the sample table at 90° in the horizontal (the direction of the polarization of the LCLS X-ray beam) to minimize elastic scattering background, and the spectrometer detector is mounted either on the sample table or on the detector robot.

## 4. Experimental geometries, endstations and scientific applications   

Serial femtosecond crystallography (SFX) has become increasingly popular since the first experiments demonstrating the method at LCLS in 2009 (Chapman *et al.*, 2011 ▸). The ability to outrun radiation damage at ambient temperature conditions has made this an appealing method for structural determination of novel structures (Redecke *et al.*, 2013 ▸) as well as revisiting cryogenic structures (Sierra *et al.*, 2015 ▸). Furthermore, the short pulses of LCLS lend themselves naturally to time-resolved studies. The majority of the experiments at the MFX instrument utilize various SFX methods, sometimes in combination with spectroscopic techniques.

The MFX instrument does not have a permanent experimental geometry. The broad expanse of the sample table and flexibility of the space allow multiple experimental geometries to be utilized. Below is an outline of different types of experimental geometries and endstations that have been interfaced to MFX along with scientific applications and results. These experimental geometries and endstations make use of the two main advantages of atmospheric-pressure operation, which are: simplified sample handling and delivery, and increased versatility of space and device choice, when vacuum is not required. For example, atmospheric-pressure operations increases the flexibility in detector selection which allows commercial detectors or detectors not custom-built for LCLS to be used. Two standard configurations are currently available to general users for fixed-target or injector-based serial crystallography.

### 4.1. Standard fixed-target serial crystallography setup   

A highly automated goniometer-based setup (Russi *et al.*, 2016 ▸) similar to those found at micro-focus crystallography beamlines at synchrotrons is made available to general users, as a collaboration between LCLS and SSRL (Cohen *et al.*, 2014 ▸). It has been optimized to provide efficient modes of serial femtosecond crystallography experiments tailored to different sample requirements and holders. Crystals can be mounted on a variety of substrates such as meshes, grids or chips affixed to standard cryo pins for robotic sample exchange and data collection at cryogenic temperatures or at room temperature in a humidity-controlled environment. An appropriate X-ray transmission can be dialed in to not obliterate large crystals or to destroy only small parts of them with a highly focused X-ray beam. Multiple modes of data collection are supported to tailor data collection to the specifics of the sample using the *Blu-Ice/DCSS* software (McPhillips *et al.*, 2002 ▸). Here, the control software of the goniometer can trigger the pulse picker to deliver single pulses of X-rays on demand. To test samples and prepare for experiments at MFX, a similar experimental environment is available at the SSRL microfocus BL12 for serial synchrotron diffraction experiments. An upgrade of the system at MFX to support 30 Hz to 120 Hz data collection rates is underway which will greatly increase experimental throughput. The SSRL goniometer setup was used at MFX to characterize the structure of a highly potent V3-glycan broadly neutralizing antibody bound to natively glycosylated HIV-1 envelope (Barnes *et al.*, 2018 ▸).

### 4.2. Standard liquid injectors   

The use of injectors to deliver protein crystal slurries into the X-ray beam is a staple of SFX experiments and a variety of injector methods are supported for general users at MFX. Although initially developed for in-vacuum operation, these liquid injectors can operate in atmospheric conditions with little to no modification. For example, high-viscosity extrusion devices used for samples in lipidic cubic phase (Weierstall *et al.*, 2014 ▸), grease matrix (Sugahara *et al.*, 2015 ▸) and other viscous media (Conrad *et al.*, 2015 ▸) can be operated at ambient pressure and are now used at synchrotron beamlines (Gati *et al.*, 2014 ▸; Nogly *et al.*, 2015 ▸; Botha *et al.*, 2015 ▸). Other injections methods such as the Microfluidic Electrokinetic Sample Holder (MESH) (Sierra *et al.*, 2012 ▸) and the gas dynamic virtual nozzle (GDVN) (DePonte *et al.*, 2008 ▸) have also been used in-air at MFX. Liquid jets in various forms are commonly used for dynamic mixing measurements (Kupitz *et al.*, 2017 ▸; Sierra *et al.*, 2015 ▸). Mix-and-probe experiments have been performed at MFX on cytochrome C oxidase (Ishigami *et al.*, 2019 ▸) and glucosyl-3-phosphoglycerate synthase enzymes which are expected to be the beginning of a growing effort in mix-and-probe SFX experiments. The MFX instrument will be an important tool for liquid-jet-based studies, crystallography or other, at LCLS. The Max Planck Institute for Medical Research in Heidelberg has built a helium enclosure called the HElium Rich Ambient system (HERA) that is now available as a standard configuration for general MFX users. Liquid jets in ambient atmospheric conditions are not subject to freezing due to evaporative cooling and less prone to precipitation; however, they result in additional background scatter from the helium or air, not present in vacuum experiments. Time-resolved pump–probe studies now represent a significant part of the MFX liquid jet operation. This trend is expected to continue with laser-activated dynamics representing an obvious use of LCLS capabilities. A planned femtosecond laser upgrade, to achieve the highest possible time resolutions, will expand the MFX capabilities further, which are now limited to nano­second dynamics.

### 4.3. RoadRunner fast scanning goniometer   

Data collection from macromolecular crystals using a fast detector operating at the full 120 Hz LCLS rate, namely the CSPAD 2.3M, was demonstrated at XPP (Roedig *et al.*, 2017 ▸). By tuning the settings of the motors, the system allows for the beam to hit regularly spaced sample windows on chips by moving at a constant speed, without stopping at every sample. Tightly spaced sample windows carry many tens of thousands of sample wells per chips, which allows for data collection rates approaching those of liquid jets. This system is now regularly deployed at MFX and can be used in combination with an optical pump laser for time-resolved studies of photo-active molecules.

### 4.4. Droplet-on-tape   

In order to improve the precision of pump–probe timing and allow for longer time delays between laser illumination and X-ray probing, a droplet-on-tape system was developed, in which droplets are acoustically ejected onto a moving polyimide belt (tape) which passes the crystal-carrying droplets under laser illumination before being probed by the X-ray beam (Fuller *et al.*, 2017 ▸). Combined with the Rayonix MX170-XFEL detector operating at 10 Hz, the system was able to efficiently probe the structure of photosystem II at different illuminated states (Young *et al.*, 2016 ▸; Fuller *et al.*, 2017 ▸; Kern *et al.*, 2018 ▸). The now-available Rayonix MX340-XFEL allows the system to operate three times faster and other future detectors that combine high speed with high dynamic range will push the data rate even further.

Simultaneous forward diffraction and X-ray emission spectra were collected for the photo-activated photosystem II while going through the Kok cycle, using the droplet-on-tape system. X-ray emission spectroscopy was used to simultaneously monitor the electronic structure with a multicrystal dispersive XES spectrometer (Alonso-Mori *et al.*, 2012 ▸) placed at 90° to collect the Mn *K*β spectrum on a shot-by-shot basis. Other published work also reports X-ray emission spectroscopy results from MFX (Fransson *et al.*, 2018 ▸), in combination with crystallography. Combining the two methods is not an idea novel to MFX but the MFX system was built having in mind this specific dual method of crystallography and spectroscopy. The use of emission spectroscopy at MFX is expected to continue to be an important use of the instrument.

**Figure 5:**
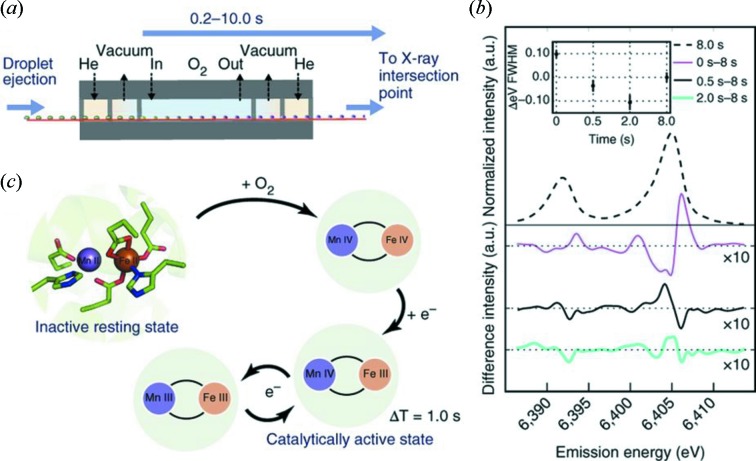
(*a*) Schematic of the differentially pumped O_2_ gas activation setup containing regions of O_2_ gas and slight negative pressure (vacuum). (*b*) The known reaction scheme of ribonucleotide reductase R2. (*c*) Emission spectra of Fe for various O_2_ exposure times. The inset shows the *K*α_1_ FWHM as a function of exposure time relative to an 8 s exposure. Reproduced with permission from Fuller *et al.* (2017 ▸).

The tape drive system can be modified, for example, to include an anaerobic-to-aerobic reaction chamber which can be used for simultaneous crystallography and spectroscopy measurements of time-resolved rapid mixing reactions. An example is shown in Fig. 5 ▸ where intermediates of ribo­nucleotide reductase R2 binding to oxygen were studied (Fuller *et al.*, 2017 ▸).

## 5. Commissioning results   

The MFX instrument was made available to users for experiments after a total of five 12-hour shifts of commissioning. The limited time available to commission the instrument allowed for characterizing the X-ray focus and verifying the X-ray optical performance. System readiness was verified via the collection of diffraction patterns from streptavidin microcrystals using the Rayonix MX170-XFEL, which demonstrated the MFX capabilities to collect high-resolution crystallographic data. The X-ray optical performance results are presented below.

The centers of all lens stacks in the transfocator were individually aligned transversely to the beam in the *X* and *Y* directions. Then the co-linearity of the *Z*-translation stage that moves the transfocator along the beam was checked by monitoring the beam centroid position at focus on a Ce:YAG screen through the full travel of 300 mm. The stage axis was manually adjusted to remove the linear component of the misalignment, leaving the non-straightness of the stage as the remaining error.

The focused beam profile was imaged using a Ce:YAG screen and long-working-distance microscope at the sample location. The beam profile was imaged while passing through the focus by moving the Ce:YAG screen and camera along the optical axis (*Z* direction). The 0.82 µm pixel size of the image on this microscope was determined using a standard resolution test chart USAF1951. Diffraction-limited calculations of an aberration-free system were also performed to estimate the modulation transfer function (MTF) of the microscope system in order to correct for the microscope resolution. This yielded a resolution of the microscope of 2.8 µm.

**Figure 6:**
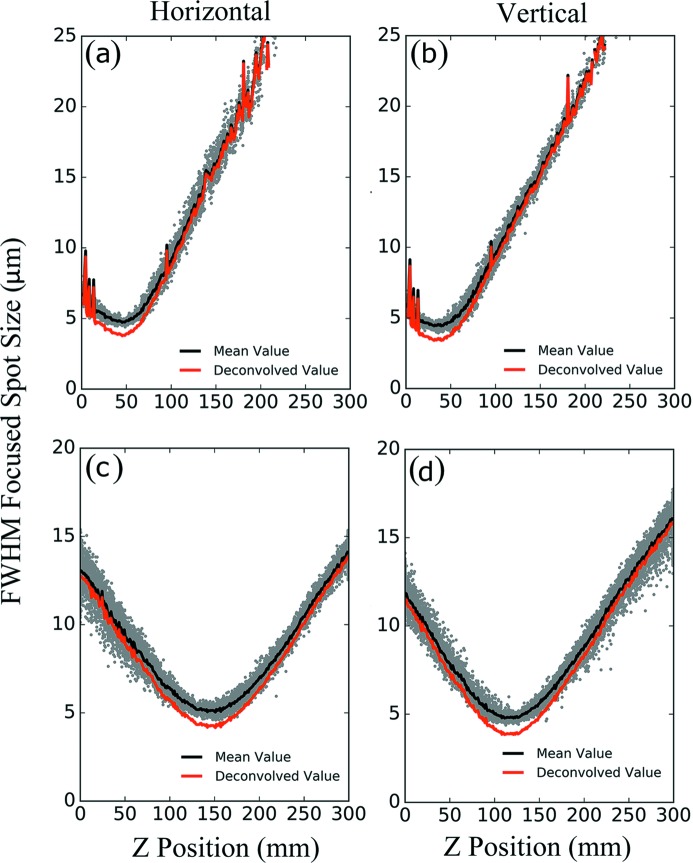
Focused beam profile characterization at 9.5 keV. The upper row represents no pre-focusing and the lower row represents pre-focusing using the lenses in the XRT. Each of the gray dots in the horizontal and vertical direction represents an LCLS shot to which a two-dimensional Gaussian profile was fitted to the measured profile. The solid black lines in (*a*), (*b*), (*c*) and (*d*) represent the average of the fitted spots within certain central transfocator *Z* position bin. (*a*) Results of horizontal spatial beam profile characterization without pre-focusing. (*b*) Results of vertical spatial beam profile of focused beam without pre-focusing. The focused beam spot size is ∼3.7 µm. The results show an astigmatic beam profile potentially due to misaligned lenses or a bending in the HOMS. (*c*) Results of horizontal spatial beam profile characterization with pre-focusing. (*d*) Results of vertical spatial beam profile characterization with pre-focusing. The results also show an obvious astigmatic beam profile possibly from misaligned (*e.g.* rotated) pre-focusing lenses.

The spatial beam profile at the source is not expected to be a perfect Gaussian distribution. Further, distortions are expected due to imperfections in the upstream hard X-ray offset mirrors system (HOMS) located in the LCLS Front End Enclosure as well at the X-ray Tunnel mirror (Pardini *et al.*, 2017 ▸). Nevertheless, a two-dimensional Gaussian fit was performed as a reasonable fit for each of the beam profile images from individual shots and the full width at half-maximum (FWHM) was extracted. The width of the fitted beam profile for both the horizontal and the vertical direction were obtained for each pulse by deconvolving the fitted Gaussian with the point-spread function (PSF) of the microscope. The PSF was determined from the known geometry of optics and performance of the optical system using the calibration target. Furthermore, to obtain an accurate focused beam spot size, uniform medium plus thin lens ray-tracing matrix analysis was employed. Based on that analysis, quadratic fitting was applied to the deconvolved two-dimensional Gaussian beam for both the horizontal and the vertical direction. Only the symmetric parts of the deconvolved curves were used to fit the through-focus profile, due to symmetry inherent in the quadratic function. From this fit, the focused beam spot size was determined. This is all shown at the X-ray energy of 9.5 keV in Fig. 6 ▸ where a beam size of 3.7 µm is obtained as an upper bound, likely limited by the resolution of the imaging system (Koch *et al.*, 1998 ▸).

**Figure 7:**
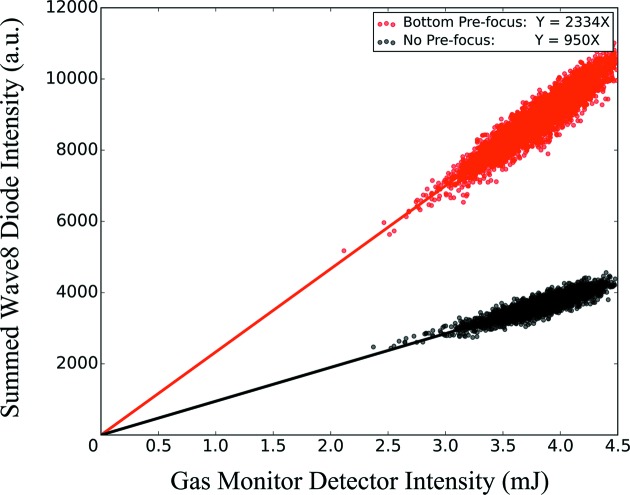
Efficiency at 7.5 keV with no pre-focusing and pre-focusing using a lens of 750 µm radius (bottom stack), the only usable pre-focusing option at 7.5 keV. Pre-focusing increases the transmission by a factor of ∼2.5. The relative single-pulse intensity measured after the focusing optics is shown as dots versus the gas detector single pulse signal, with a linear fit as a line. The slope represents the relative efficiency.

As previously mentioned, MFX has the capability to pre-focus the beam with CRLs located in the XRT. This can increase the transmission of the beamline, at the cost of an increased focal spot size due to the effective source being closer in a pre-focusing geometry. The transmission gain via pre-focusing of the beam was measured and is shown in Fig. 7 ▸. This was measured by setting the slits to match the clear aperture of the primary focusing lenses and measuring the transmission through these slits with and without the pre-focusing lenses. Too small a pre-focusing lens radius leads to too strong a pre-focusing and a beam that is thus too small at the transfocator, which cannot be used. Therefore, pre-focusing was characterized only for pre-focusing lens stacks that lead to a usable situation, ideally producing a beam at the transfocator that matches the CRL clear aperture. The LCLS beam becomes larger for lower photon energy. Therefore, larger gains in efficiency from pre-focusing are expected at lower photon energies.

The transmitted beam intensity was measured using a modified version of the single-shot intensity–position monitor (Feng *et al.*, 2011 ▸). This modified version, called Wave8, measures back-scattering from a thin transmissive Si_3_N_4_ target using eight diodes and shows good linearity with the LCLS gas monitor. By comparing the Wave8 measurements with the upstream gas monitor detector, the transmission efficiency can be evaluated under no pre-focusing and pre-focusing conditions. Pulse intensities under 2 mJ were omitted from the analysis. Linear fitting was applied to each of the scatter plots in Fig. 7 ▸.

## 6. Conclusion   

The Macromolecular Femtosecond Crystallography (MFX) instrument at LCLS can perform a wide variety of X-ray crystallography experiments in the hard X-ray regime (5–13 keV today and 5–25 keV with the upcoming LCLS-II upgrade) and takes advantage of the unique capabilities of X-ray free-electron lasers. It is primarily suitable for experiments which require high fluence, that may not be compatible with the challenges of using a vacuum environment. This instrument allows for a continued development of femto­second structural biology with radiation-damage-free structure determination at room temperature. As the MFX instrument continues to develop, new opportunities for optically induced and chemically induced time-resolved experiments will arise. Advancements in automation to the sample delivery systems and detector technology will improve the efficiency of data collection, which, in combination with potential multiplexing, will increase the output of science and number of user hours the facility can support for biological imaging experiments. MFX is a versatile system suitable for creative and unique experimental geometries not limited to macromolecular crystallography. More details about the MFX instrument can be found at https://lcls.slac.stanford.edu/mfx.

## 7. Facility access   

LCLS instruments are open to academia, industry, government agencies and research institutes worldwide for scientific investigations. There are typically two calls for proposals per year and an external peer-review committee evaluates proposals based on scientific merit and instrument suitability. There are also typically calls for shorter protein crystal screening (PCS) beam times, which may request either the standard CXI or standard MFX endstations. Access is without charge for users who intend to publish their results. The facility is equipped to assist in all aspects of the experiment, from planning, sample preparation and delivery, to data collection and analysis. Prospective users are encouraged to contact instrument staff members to learn more about the science and capabilities of the facility, and opportunities for collaboration.

## Figures and Tables

**Table 1:** X-ray parameters and capabilities of the MFX instrument Existing LCLS parameters are derived from Emma *et al.* (2010 ▸), Amann *et al.* (2012 ▸), Bostedt *et al.* (2016 ▸) and Liu *et al.* (2018 ▸). LCLS-II capabilities are extracted from Galayda (2014 ▸).

Instrument name	MFX
Flat mirrors, incidence angle	2 × B_4_C or Ni on Si, 2.1 mrad, located in the Front End Enclosure and 1 × B_4_C or Rh on Si, 2.75 mrad, located in the X-ray Tunnel
Monochromaticity (Δ*E*/*E*)†	1–3 × 10^−3^ (SASE); 2 × 10^−4^ (seeded)
Energy range (keV)	5–25‡ (fundamental)
Unfocused beam size (µm)	800 @ 8.3 keV
Focused beam size (µm)	2–500 (3.7 measured as smallest focus)
Focusing optics	Be lenses, 2D focusing; transfocator
Flux (photons pulse^−1^)	∼1 × 10^12^ (fundamental)§
Pulse energy (mJ pulse^−1^)	2–4 (fundamental at the source)
Pulse length (fs)	5–120, 30 nominal
Repetition rate (Hz)	120, 60, <30, on demand
Standard detectors	CSPAD2.3M, CSPAD-140k, ePix100, Jungfrau 0.5M, Jungfrau 1M, Rayonix MX170-XFEL (discontinued), Rayonix MX340-XFEL
Sample delivery	Sample table with six degrees of freedom; liquid jets (He and atmospheric enclosure); fixed-target fast-scan chips; droplet-on-tape; cryo- and room-temparature, goniometer with robotic sample mounting

† Typical single-shot value.

‡ 13 keV before LCLS-II upgrade.

§ Beamline and instrument transmission is typically ∼50–75%.

## References

[bb1] Alonso-Mori, R., Kern, J., Gildea, R. J., Sokaras, D., Weng, T.-C., Lassalle-Kaiser, B., Tran, R., Hattne, J., Laksmono, H., Hellmich, J., Glöckner, C., Echols, N., Sierra, R. G., Schafer, D. W., Sellberg, J., Kenney, C., Herbst, R., Pines, J., Hart, P., Herrmann, S., Grosse-Kunstleve, R. W., Latimer, M. J., Fry, A. R., Messerschmidt, M. M., Miahnahri, A., Seibert, M. M., Zwart, P. H., White, W. E., Adams, P. D., Bogan, M. J., Boutet, S., Williams, G. J., Zouni, A., Messinger, J., Glatzel, P., Sauter, N. K., Yachandra, V. K., Yano, J. & Bergmann, U. (2012). *Proc. Natl Acad. Sci. USA*, **109**, 19103–19107.

[bb2] Amann, J., and Berg, W. B. V., Decker, F.-J., Ding, Y., Emma, P., Feng, Y., Frisch, J., Fritz, D., Hastings, J., Huang, Z., Krzywinski, J., Lindberg, R., Loos, H., Lutman, A., Nuhn, H.-D., Ratner, D., Rzepiela, J., Shu, D., Shvyd’ko, Y., Spampinati, S., Stoupin, S., Terentyev, S., Trakhtenberg, E., Walz, D., Welch, J., Wu, J., Zholents, A. & Zhu, D. (2012). *Nat. Photon.* **6**, 693.

[bb3] Barnes, C. O., Gristick, H. B., Freund, N. T., Escolano, A., Lyubimov, A. Y., Hartweger, H., West, A. P., Cohen, A. E., Nussenzweig, M. C. & Bjorkman, P. J. (2018). *Nat. Commun.* **9**, 1251. 10.1038/s41467-018-03632-y PMC5871869 29593217

[bb51] Blaj, G., Dragone, A., Kenney, C. J., Abu-Nimeh, F., Caragiulo, P., Doering, D., Kwiatkowski, M., Markovic, B., Pines, J., Weaver, M., Boutet, S., Carini, G., Chang, C.-E., Hart, P., Hasi, J., Hayes, M., Herbst, R., Koglin, J., Nakahara, K., Segal, J. & Haller, G. (2019). *AIP Conf. Proc.* **2054**, 060062.

[bb4] Bostedt, C., Boutet, S., Fritz, D. M., Huang, Z., Lee, H. J., Lemke, H. T., Robert, A., Schlotter, W. F., Turner, J. J. & Williams, G. J. (2016). *Rev. Mod. Phys.* **88**, 015007.

[bb5] Botha, S., Nass, K., Barends, T. R. M., Kabsch, W., Latz, B., Dworkowski, F., Foucar, L., Panepucci, E., Wang, M., Shoeman, R. L., Schlichting, I. & Doak, R. B. (2015). *Acta Cryst.* D**71**, 387–397. 10.1107/S1399004714026327 25664750

[bb6] Boutet, S., Cohen, A. E. & Wakatsuki, S. (2016). *Synchrotron Radiat. News*, **29**(1), 23–28. 10.1080/08940886.2016.1124681 PMC5519296 28736484

[bb7] Boutet, S., Fromme, P. & Hunter, M. S. (2018). *X-ray Free Electron Lasers — A Revolution in Structural Biology*, 1st ed. Springer International Publishing.

[bb8] Carini, G. A., Alonso-Mori, R., Blaj, G., Caragiulo, P., Chollet, M., Damiani, D., Dragone, A., Feng, Y., Haller, G., Hart, P., Hasi, J., Herbst, R., Herrmann, S., Kenney, C., Lemke, H., Manger, L., Markovic, B., Mehta, A., Nelson, S., Nishimura, K., Osier, S., Pines, J., Reese, B., Robert, A., Segal, J., Sikorski, M., Song, S., Thayer, J., Tomada, A., Weaver, M. & Zhu, D. (2016). *AIP Conf. Proc.* **1741**, 040008.

[bb9] Chapman, H. N., Fromme, P., Barty, A., White, T., Kirian, R. A., Aquila, A., Hunter, M. S., Schulz, J., DePonte, D. P., Weierstall, U., Doak, R. B., Maia, F. R. N. C., Martin, A. V., Schlichting, I., Lomb, L., Coppola, N., Shoeman, R. L., Epp, S. W., Hartmann, R., Rolles, D., Rudenko, A., Foucar, L., Kimmel, N., Weidenspointner, G., Holl, P., Liang, M., Barthelmess, M., Caleman, C., Boutet, S., Bogan, M. J., Krzywinski, J., Bostedt, C., Bajt, S., Gumprecht, L., Rudek, B., Erk, B., Schmidt, C., Hömke, A., Reich, C., Pietschner, D., Strüder, L., Hauser, G., Gorke, H., Ullrich, J., Herrmann, S., Schaller, G., Schopper, F., Soltau, H., Kühnel, K., Messerschmidt, M., Bozek, J. D., Hau-Riege, S. P., Frank, M., Hampton, C. Y., Sierra, R. G., Starodub, D., Williams, G. J., Hajdu, J., Timneanu, N., Seibert, M. M., Andreasson, J., Rocker, A., Jönsson, O., Svenda, M., Stern, S., Nass, K., Andritschke, R., Schröter, C., Krasniqi, F., Bott, M., Schmidt, K. E., Wang, X., Grotjohann, I., Holton, J. M., Barends, T. R. M., Neutze, R., Marchesini, S., Fromme, R., Schorb, S., Rupp, D., Adolph, M., Gorkhover, T., Andersson, I., Hirsemann, H., Potdevin, G., Graafsma, H., Nilsson, B. & Spence, J. C. H. (2011). *Nature*, **470**, 73–77.

[bb10] Chollet, M., Alonso-Mori, R., Cammarata, M., Damiani, D., Defever, J., Delor, J. T., Feng, Y., Glownia, J. M., Langton, J. B., Nelson, S., Ramsey, K., Robert, A., Sikorski, M., Song, S., Stefanescu, D., Srinivasan, V., Zhu, D., Lemke, H. T. & Fritz, D. M. (2015). *J. Synchrotron Rad.* **22**, 503–507. 10.1107/S1600577515005135 PMC4416667 25931060

[bb11] Clark, J. N., Beitra, L., Xiong, G., Higginbotham, A., Fritz, D. M., Lemke, H. T., Zhu, D., Chollet, M., Williams, G. J., Messerschmidt, M., Abbey, B., Harder, R. J., Korsunsky, A. M., Wark, J. S. & Robinson, I. K. (2013). *Science*, **341**, 56–59. 10.1126/science.1236034 23704372

[bb12] Cohen, A. E., Soltis, S. M., González, A., Aguila, L., Alonso-Mori, R., Barnes, C. O., Baxter, E. L., Brehmer, W., Brewster, A. S., Brunger, A. T., Calero, G., Chang, J. F., Chollet, M., Ehrensberger, P., Eriksson, T. L., Feng, Y., Hattne, J., Hedman, B., Hollenbeck, M., Holton, J. M., Keable, S., Kobilka, B. K., Kovaleva, E. G., Kruse, A. C., Lemke, H. T., Lin, G., Lyubimov, A. Y., Manglik, A., Mathews, I. I., McPhillips, S. E., Nelson, S., Peters, J. W., Sauter, N. K., Smith, C., Song, J., Stevenson, H. P., Tsai, Y., Uervirojnangkoorn, M., Vinetsky, V., Wakatsuki, S., Weis, W. I., Zadvornyy, O. A., Zeldin, O. B., Zhu, D. & Hodgson, K. O. (2014). *Proc. Natl Acad. Sci.* **111**, 17122–17127. 10.1073/pnas.1418733111 PMC4260607 25362050

[bb13] Conrad, C. E., Basu, S., James, D., Wang, D., Schaffer, A., Roy-Chowdhury, S., Zatsepin, N. A., Aquila, A., Coe, J., Gati, C., Hunter, M. S., Koglin, J. E., Kupitz, C., Nelson, G., Subramanian, G., White, T. A., Zhao, Y., Zook, J., Boutet, S., Cherezov, V., Spence, J. C. H., Fromme, R., Weierstall, U. & Fromme, P. (2015). *IUCrJ*, **2**, 421–430. 10.1107/S2052252515009811 PMC4491314 26177184

[bb14] DePonte, D. P., Weierstall, U., Schmidt, K., Warner, J., Starodub, D., Spence, J. C. H. & Doak, R. B. (2008). *J. Phys. D Appl. Phys.* **41**, 195505.

[bb15] Emma, P., Akre, R., Arthur, J., Bionta, R., Bostedt, C., Bozek, J., Brachmann, A., Bucksbaum, P., Coffee, R., Decker, F.-J., Ding, Y., Dowell, D., Edstrom, S., Fisher, A., Frisch, J., Gilevich, S., Hastings, J., Hays, G., Hering, P., Huang, Z., Iverson, R., Loos, H., Messerschmidt, M., Miahnahri, A., Moeller, S., Nuhn, H.-D., Pile, G., Ratner, D., Rzepiela, J., Schultz, D., Smith, T., Stefan, P., Tompkins, H., Turner, J., Welch, J., White, W., Wu, J., Yocky, G. & Galayda, J. (2010). *Nat. Photon.* **4**, 641–647.

[bb16] Feng, Y., Alonso-Mori, R., Barends, T. R. M., Blank, V. D., Botha, S., Chollet, M., Damiani, D. S., Doak, R. B., Glownia, J. M., Koglin, J. M., Lemke, H. T., Messerschmidt, M., Nass, K., Nelson, S., Schlichting, I., Shoeman, R. L., Shvyd’ko, Y. V., Sikorski, M., Song, S., Stoupin, S., Terentyev, S., Williams, G. J., Zhu, D., Robert, A. & Boutet, S. (2015). *J. Synchrotron Rad.* **22**, 626–633. 10.1107/S1600577515003999 PMC4416679 25931078

[bb17] Feng, Y., Feldkamp, J. M., Fritz, D. M., Cammarata, M., Aymeric, R., Caronna, C., Lemke, H. T., Zhu, D., Lee, S., Boutet, S., Williams, G., Tono, K., Yabashi, M. & Hastings, J. B. (2011). *Proc. SPIE*, **8140**, 81400Q.

[bb18] Fransson, T., Chatterjee, R., Fuller, F. D., Gul, S., Weninger, C., Sokaras, D., Kroll, T., Alonso-Mori, R., Bergmann, U., Kern, J., Yachandra, V. K. & Yano, J. (2018). *Biochemistry*, **57**, 4629–4637. 10.1021/acs.biochem.8b00325 PMC6081253 29906115

[bb19] Fuller, F. D., Gul, S., Chatterjee, R., Burgie, E. S., Young, I. D., Lebrette, H., Srinivas, V., Brewster, A. S., Michels-Clark, T., Clinger, J. A., Andi, B., Ibrahim, M., Pastor, E., de Lichtenberg, C., Hussein, R., Pollock, C. J., Zhang, M., Stan, C. A., Kroll, T., Fransson, T., Weninger, C., Kubin, M., Aller, P., Lassalle, L., Bräuer, P., Miller, M. D., Amin, M., Koroidov, S., Roessler, C. G., Allaire, M., Sierra, R. G., Docker, P. T., Glownia, J. M., Nelson, S., Koglin, J. E., Zhu, D., Chollet, M., Song, S., Lemke, H., Liang, M., Sokaras, D., Alonso-Mori, R., Zouni, A., Messinger, J., Bergmann, U., Boal, A. K., Bollinger, J. M., Krebs, C., Högbom, M., Phillips, G. N., Vierstra, R. D., Sauter, N. K., Orville, A. M., Kern, J., Yachandra, V. K. & Yano, J. (2017). *Nat. Methods*, **14**, 443–449. 10.1038/nmeth.4195 PMC5376230 28250468

[bb20] Galayda, J. (2014). *Proceedings of the 5th International Particle Accelerator Conference (IPAC-2014)*, 15–20 June 2014, Dresden, Germany, pp. 935–937. TUOCA01.

[bb21] Gati, C., Bourenkov, G., Klinge, M., Rehders, D., Stellato, F., Oberthür, D., Yefanov, O., Sommer, B. P., Mogk, S., Duszenko, M., Betzel, C., Schneider, T. R., Chapman, H. N. & Redecke, L. (2014). *IUCrJ*, **1**, 87–94. 10.1107/S2052252513033939 PMC4062088 25075324

[bb22] Harmand, M., Coffee, R., Bionta, M. R., Chollet, M., French, D., Zhu, D., Fritz, D. M., Lemke, H. T., Medvedev, N., Ziaja, B., Toleikis, S. & Cammarata, M. (2013). *Nat. Photon.* **7**, 215–218.

[bb23] Hart, P., Boutet, S., Carini, G., Dubrovin, M., Duda, B., Fritz, D., Haller, G., Herbst, R., Herrmann, S., Kenney, C., Kurita, N., Lemke, H., Messerschmidt, M., Nordby, M., Pines, J., Schafer, D., Swift, M., Weaver, M., Williams, G., Zhu, D., Van Bakel, N. & Morse, J. (2012). *Proc. SPIE*, **8504**, 85040C.

[bb50] Ishigami, I., Lewis-Ballester, A., Echelmeier, A., Brehm, G., Zatsepin, N. A., Grant, T. D., Coe, J. D., Lisova, S., Nelson, G., Zhang, S., Dobson, Z. F., Boutet, S., Sierra, R. G., Batyuk, A., Fromme, P., Fromme, R., Spence, J. C. H., Ros, A., Yeh, S.-R. & Rousseau, D. L. (2019). *Proc. Natl Acad. Sci. USA*, doi:10.1073/pnas.1814526116. 10.1073/pnas.1814526116 PMC6397517 30808749

[bb24] Kern, J., Alonso-Mori, R., Tran, R., Hattne, J., Gildea, R. J., Echols, N., Glöckner, C., Hellmich, J., Laksmono, H., Sierra, R. G., Lassalle-Kaiser, B., Koroidov, S., Lampe, A., Han, G., Gul, S., Difiore, D., Milathianaki, D., Fry, A. R., Miahnahri, A., Schafer, D. W., Messerschmidt, M., Seibert, M. M., Koglin, J. E., Sokaras, D., Weng, T.-C., Sellberg, J., Latimer, M. J., Grosse-Kunstleve, R. W., Zwart, P. H., White, W. E., Glatzel, P., Adams, P. D., Bogan, M. J., Williams, G. J., Boutet, S., Messinger, J., Zouni, A., Sauter, N. K., Yachandra, V. K., Bergmann, U. & Yano, J. (2013). *Science*, **340**, 491–495.

[bb25] Kern, J., Chatterjee, R., Young, I. D., Fuller, F. D., Lassalle, L., Ibrahim, M., Gul, S., Fransson, T., Brewster, A. S., Alonso-Mori, R., Hussein, R., Zhang, M., Douthit, L., de Lichtenberg, C., Cheah, M. H., Shevela, D., Wersig, J., Seuffert, I., Sokaras, D., Pastor, E., Weninger, C., Kroll, T., Sierra, R. G., Aller, P., Butryn, A., Orville, A. M., Liang, M., Batyuk, A., Koglin, J. E., Carbajo, S., Boutet, S., Moriarty, N. W., Holton, J. M., Dobbek, H., Adams, P. D., Bergmann, U., Sauter, N. K., Zouni, A., Messinger, J., Yano, J. & Yachandra, V. K. (2018). *Nature*, **563**, 421–425.

[bb26] Koch, A., Raven, C., Spanne, P. & Snigirev, A. (1998). *J. Opt. Soc. Am. A*, **15**, 1940.

[bb27] Kupitz, C., Basu, S., Grotjohann, I., Fromme, R., Zatsepin, N. A., Rendek, K. N., Hunter, M. S., Shoeman, R. L., White, T. A., Wang, D., James, D., Yang, J.-H., Cobb, D. E., Reeder, B., Sierra, R. G., Liu, H., Barty, A., Aquila, A. L., Deponte, D., Kirian, R. A., Bari, S., Bergkamp, J. J., Beyerlein, K. R., Bogan, M. J., Caleman, C., Chao, T.-C., Conrad, C. E., Davis, K. M., Fleckenstein, H., Galli, L., Hau-Riege, S. P., Kassemeyer, S., Laksmono, H., Liang, M., Lomb, L., Marchesini, S., Martin, A. V., Messerschmidt, M., Milathianaki, D., Nass, K., Ros, A., Roy-Chowdhury, S., Schmidt, K., Seibert, M., Steinbrener, J., Stellato, F., Yan, L., Yoon, C., Moore, T. A., Moore, A. L., Pushkar, Y., Williams, G. J., Boutet, S., Doak, R. B., Weierstall, U., Frank, M., Chapman, H. N., Spence, J. C. H. & Fromme, P. (2014). *Nature*, **513**, 261–265.

[bb28] Kupitz, C., Olmos, J. L., Holl, M., Tremblay, L., Pande, K., Pandey, S., Oberthür, D., Hunter, M., Liang, M., Aquila, A., Tenboer, J., Calvey, G., Katz, A., Chen, Y., Wiedorn, M. O., Knoska, J., Meents, A., Majriani, V., Norwood, T., Poudyal, I., Grant, T., Miller, M. D., Xu, W., Tolstikova, A., Morgan, A., Metz, M., Martin-Garcia, J. M., Zook, J. D., Roy-Chowdhury, S., Coe, J., Nagaratnam, N., Meza, D., Fromme, R., Basu, S., Frank, M., White, T., Barty, A., Bajt, S., Yefanov, O., Chapman, H. N., Zatsepin, N., Nelson, G., Weierstall, U., Spence, J., Schwander, P., Pollack, L., Fromme, P., Ourmazd, A., Phillips, G. N. Jr & Schmidt, M. (2017). *Struct. Dyn.* **4**, 044003. 10.1063/1.4972069 PMC5178802 28083542

[bb29] Liang, M., Williams, G. J., Messerschmidt, M., Seibert, M. M., Montanez, P. A., Hayes, M., Milathianaki, D., Aquila, A., Hunter, M. S., Koglin, J. E., Schafer, D. W., Guillet, S., Busse, A., Bergan, R., Olson, W., Fox, K., Stewart, N., Curtis, R., Miahnahri, A. A. & Boutet, S. (2015). *J. Synchrotron Rad.* **22**, 514–519. 10.1107/S160057751500449X PMC4416669 25931062

[bb30] Liu, Y., Seaberg, M., Zhu, D., Krzywinski, J., Seiboth, F., Hardin, C., Cocco, D., Aquila, A., Nagler, B., Lee, H. J., Boutet, S., Feng, Y., Ding, Y., Marcus, G. & Sakdinawat, A. (2018). *Optica*, **5**, 967–975.

[bb32] McPhillips, T. M., McPhillips, S. E., Chiu, H.-J., Cohen, A. E., Deacon, A. M., Ellis, P. J., Garman, E., Gonzalez, A., Sauter, N. K., Phizackerley, R. P., Soltis, S. M. & Kuhn, P. (2002). *J. Synchrotron Rad.* **9**, 401–406. 10.1107/s0909049502015170 12409628

[bb31] Marinelli, A., Ratner, D., Lutman, A., Turner, J., Welch, J., Decker, F. J., Loos, H., Behrens, C., Gilevich, S., Miahnahri, A. A., Vetter, S., Maxwell, T. J., Ding, Y., Coffee, R., Wakatsuki, S. & Huang, Z. (2015). *Nat. Commun.* **6**, 6369. 10.1038/ncomms7369 PMC4366525 25744344

[bb33] Minitti, M. P., Robinson, J. S., Coffee, R. N., Edstrom, S., Gilevich, S., Glownia, J. M., Granados, E., Hering, P., Hoffmann, M. C., Miahnahri, A., Milathianaki, D., Polzin, W., Ratner, D., Tavella, F., Vetter, S., Welch, M., White, W. E. & Fry, A. R. (2015). *J. Synchrotron Rad.* **22**, 528–531. 10.1107/S1600577515006244 PMC4416671 25931064

[bb34] Nagler, B., Arnold, B., Bouchard, G., Boyce, R. F., Boyce, R. M., Callen, A., Campell, M., Curiel, R., Galtier, E., Garofoli, J., Granados, E., Hastings, J., Hays, G., Heimann, P., Lee, R. W., Milathianaki, D., Plummer, L., Schropp, A., Wallace, A., Welch, M., White, W., Xing, Z., Yin, J., Young, J., Zastrau, U. & Lee, H. J. (2015). *J. Synchrotron Rad.* **22**, 520–525. 10.1107/S1600577515004865 PMC4416670 25931063

[bb35] Neutze, R., Wouts, R., van der Spoel, D., Weckert, E. & Hajdu, J. (2000). *Nature*, **406**, 752–757. 10.1038/35021099 10963603

[bb36] Nogly, P., James, D., Wang, D., White, T. A., Zatsepin, N., Shilova, A., Nelson, G., Liu, H., Johansson, L., Heymann, M., Jaeger, K., Metz, M., Wickstrand, C., Wu, W., Båth, P., Berntsen, P., Oberthuer, D., Panneels, V., Cherezov, V., Chapman, H., Schertler, G., Neutze, R., Spence, J., Moraes, I., Burghammer, M., Standfuss, J. & Weierstall, U. (2015). *IUCrJ*, **2**, 168–176. 10.1107/S2052252514026487 PMC4392771 25866654

[bb37] Pardini, T., Aquila, A., Boutet, S., Cocco, D. & Hau-Riege, S. P. (2017). *J. Synchrotron Rad.* **24**, 738–743. 10.1107/S1600577517007032 PMC5493023 28664879

[bb38] Redecke, L., Nass, K., DePonte, D. P., White, T. A., Rehders, D., Barty, A., Stellato, F., Liang, M., Barends, T. R. M., Boutet, S., Williams, G. J., Messerschmidt, M., Seibert, M. M., Aquila, A., Arnlund, D., Bajt, S., Barth, T., Bogan, M. J., Caleman, C., Chao, T.-C., Doak, R. B., Fleckenstein, H., Frank, M., Fromme, R., Galli, L., Grotjohann, I., Hunter, M. S., Johansson, L. C., Kassemeyer, S., Katona, G., Kirian, R. A., Koopmann, R., Kupitz, C., Lomb, L., Martin, A. V., Mogk, S., Neutze, R., Shoeman, R. L., Steinbrener, J., Timneanu, N., Wang, D., Weierstall, U., Zatsepin, N. A., Spence, J. C. H., Fromme, P., Schlichting, I., Duszenko, M., Betzel, C. & Chapman, H. N. (2013). *Science*, **339**, 227–230.

[bb39] Roedig, P., Ginn, H. M., Pakendorf, T., Sutton, G., Harlos, K., Walter, T. S., Meyer, J., Fischer, P., Duman, R., Vartiainen, I., Reime, B., Warmer, M., Brewster, A. S., Young, I. D., Michels-Clark, T., Sauter, N. K., Kotecha, A., Kelly, J., Rowlands, D. J., Sikorsky, M., Nelson, S., Damiani, D. S., Alonso-Mori, R., Ren, J., Fry, E. E., David, C., Stuart, D. I., Wagner, A. & Meents, A. (2017). *Nat. Methods* **14**, 805–810. 10.1038/nmeth.4335 PMC5588887 28628129

[bb40] Russi, S., Song, J., McPhillips, S. E. & Cohen, A. E. (2016). *J. Appl. Cryst.* **49**, 622–626. 10.1107/S1600576716000649 PMC4815877 27047309

[bb41] Sierra, R. G., Gati, C., Laksmono, H., Dao, E. H., Gul, S., Fuller, F., Kern, J., Chatterjee, R., Ibrahim, M., Brewster, A. S., Young, I. D., Michels-Clark, T., Aquila, A., Liang, M., Hunter, M. S., Koglin, J. E., Boutet, S., Junco, E. A., Hayes, B., Bogan, M. J., Hampton, C. Y., Puglisi, E. V., Sauter, N. K., Stan, C. A., Zouni, A., Yano, J., Yachandra, V. K., Soltis, S. M., Puglisi, J. D. & DeMirci, H. (2015). *Nat. Methods*, **13**, 59–62. 10.1038/nmeth.3667 PMC4890631 26619013

[bb42] Sierra, R. G., Laksmono, H., Kern, J., Tran, R., Hattne, J., Alonso-Mori, R., Lassalle-Kaiser, B., Glöckner, C., Hellmich, J., Schafer, D. W., Echols, N., Gildea, R. J., Grosse-Kunstleve, R. W., Sellberg, J., McQueen, T. A., Fry, A. R., Messerschmidt, M. M., Miahnahri, A., Seibert, M. M., Hampton, C. Y., Starodub, D., Loh, N. D., Sokaras, D., Weng, T.-C., Zwart, P. H., Glatzel, P., Milathianaki, D., White, W. E., Adams, P. D., Williams, G. J., Boutet, S., Zouni, A., Messinger, J., Sauter, N. K., Bergmann, U., Yano, J., Yachandra, V. K. & Bogan, M. J. (2012). *Acta Cryst.* D**68**, 1584–1587.

[bb43] Sugahara, M., Mizohata, E., Nango, E., Suzuki, M., Tanaka, T., Masuda, T., Tanaka, R., Shimamura, T., Tanaka, Y., Suno, C., Ihara, K., Pan, D., Kakinouchi, K., Sugiyama, S., Murata, M., Inoue, T., Tono, K., Song, C., Park, J., Kameshima, T., Hatsui, T., Joti, Y., Yabashi, M. & Iwata, S. (2015). *Nat. Methods*, **12**, 61–63. 10.1038/nmeth.3172 25384243

[bb44] Tono, K., Nango, E., Sugahara, M., Song, C., Park, J., Tanaka, T., Tanaka, R., Joti, Y., Kameshima, T., Ono, S., Hatsui, T., Mizohata, E., Suzuki, M., Shimamura, T., Tanaka, Y., Iwata, S. & Yabashi, M. (2015). *J. Synchrotron Rad.* **22**, 532–537. 10.1107/S1600577515004464 PMC4817517 25931065

[bb45] Vaughan, G. B. M., Wright, J. P., Bytchkov, A., Rossat, M., Gleyzolle, H., Snigireva, I. & Snigirev, A. (2011). *J. Synchrotron Rad.* **18**, 125–133. 10.1107/S0909049510044365 PMC3267637 21335897

[bb46] Weierstall, U., James, D., Wang, C., White, T., Wang, D., Liu, W., Spence, J. C. H., Bruce Doak, R., Nelson, G., Fromme, P., Fromme, P., Grotjohann, I., Kupitz, C., Zatsepin, N. A., Liu, H., Basu, S., Wacker, D., Won Han, G., Katritch, V., Boutet, S., Messerschmidt, M., Williams, G. J., Koglin, J. E., Marvin Seibert, M., Klinker, M., Gati, C., Shoeman, R. L., Barty, A., Chapman, H. N., Kirian, R. A., Beyerlein, K. R., Stevens, R. C., Li, D., Shah, S. T. A., Howe, N., Caffrey, M. & Cherezov, V. (2014). *Nat. Commun.* **5**, 3309. 10.1038/ncomms4309 PMC4061911 24525480

[bb47] White, W. E., Robert, A. & Dunne, M. (2015). *J. Synchrotron Rad.* **22**, 472–476. 10.1107/S1600577515005196 PMC4416663 25931055

[bb48] Young, I. D., Ibrahim, M., Chatterjee, R., Gul, S., Fuller, F. D., Koroidov, S., Brewster, A. S., Tran, R., Alonso-Mori, R., Kroll, T., Michels-Clark, T., Laksmono, H., Sierra, R. G., Stan, C. A., Hussein, R., Zhang, M., Douthit, L., Kubin, M., de Lichtenberg, C., Vo Pham, L., Nilsson, H., Cheah, M. H., Shevela, D., Saracini, C., Bean, M. A., Seuffert, I., Sokaras, D., Weng, T.-C., Pastor, E., Weninger, C., Fransson, T., Lassalle, L., Bräuer, P., Aller, P., Docker, P. T., Andi, B., Orville, A. M., Glownia, J. M., Nelson, S., Sikorski, M., Zhu, D., Hunter, M. S., Lane, T. J., Aquila, A., Koglin, J. E., Robinson, J., Liang, M., Boutet, S., Lyubimov, A. Y., Uervirojnangkoorn, M., Moriarty, N. W., Liebschner, D., Afonine, P. V., Waterman, D. G., Evans, G., Wernet, P., Dobbek, H., Weis, W. I., Brunger, A. T., Zwart, P. H., Adams, P. D., Zouni, A., Messinger, J., Bergmann, U., Sauter, N. K., Kern, J., Yachandra, V. K. & Yano, J. (2016). *Nature*, **540**, 453–457.

[bb49] Zhu, D., Cammarata, M., Feldkamp, J. M., Fritz, D. M., Hastings, J. B., Lee, S., Lemke, H. T., Robert, A., Turner, J. L. & Feng, Y. (2012). *Appl. Phys. Lett.* **101**, 034103.

